# Hybrid nanostructured coating for increased resistance of prosthetic devices to staphylococcal colonization

**DOI:** 10.1186/1556-276X-8-6

**Published:** 2013-01-02

**Authors:** Ion Anghel, Alexandru Mihai Grumezescu

**Affiliations:** 1 ENT, “Carol Davila” University of Medicine and Pharmacy, Traian Vuia no.6, Bucharest 020956, Romania; 2 Doctor Anghel Medical Center, Theodor Sperantia Street, Bucharest, 30932, Romania; 3 Department of Science and Engineering of Oxidic Materials and Nanomaterials, Faculty of Applied Chemistry and Materials Science, University Politehnica of Bucharest, Polizu Street no 1-7, Bucharest 011061, Romania

## Abstract

Prosthetic medical device-associated infections are responsible for significant morbidity and mortality rates. Novel improved materials and surfaces exhibiting inappropriate conditions for microbial development are urgently required in the medical environment. This study reveals the benefit of using natural *Mentha piperita* essential oil, combined with a 5 nm core/shell nanosystem-improved surface exhibiting anti-adherence and antibiofilm properties. This strategy reveals a dual role of the nano-oil system; on one hand, inhibiting bacterial adherence and, on the other hand, exhibiting bactericidal effect, the core/shell nanosystem is acting as a controlled releasing machine for the essential oil. Our results demonstrate that this dual nanobiosystem is very efficient also for inhibiting biofilm formation, being a good candidate for the design of novel material surfaces used for prosthetic devices.

## Background

*Staphylococcus aureus* was recognized as a major pathogen soon after its discovery in the late nineteenth century. This organism causes a broad range of conditions, ranging from asymptomatic colonization to severe invasive infections which can progress to complicated septicemia, osteomyelitis, septic arthritis, or endocarditis [1,2]. *S. aureus* is a major cause of nosocomial infections and is responsible for significant morbidity, mortality, and an extended hospital stay [3,4]. This Gram-positive bacterium possesses specific surface proteins such as fibronectin-binding proteins, collagen-binding proteins, and fibrinogen-binding proteins, which have been implicated as mediators in specific bacterial binding to the extracellular matrix and subsequent biofilm development [1,5-7].

The increased use of prosthetic devices during the past decades has been accompanied by a constantly increased number of prosthetic device infections [8]. *S. aureus* is a widespread bacterium, being found on the skin and mucosa of healthy persons; therefore, prosthesis-associated infections incriminating this pathogen are frequently encountered [9]. Prosthesis-associated infections could be the results of microbial colonization by three routes: (a) direct inoculation at the time of implantation, (b) hematogenous spreading during bacteremia, or (c) direct contiguous spreading from an adjacent infectious focus [10].

One of the most severe complications is a biofilm-associated infection of a prosthetic device due to the fact that biofilm bacteria are different from planktonic cells, being usually more resistant. The biofilm cells are resistant to all kinds of antimicrobial substances: antibiotics, antiseptics, disinfectants; this kind of resistance, consecutive to biofilm formation, is phenotypic, behavioral, and more recently, called tolerance [43,44].

Among the promising approaches to combat biofilm infections is the generation of surface modification of devices to reduce microbial attachment and biofilm development as well as incorporation of antimicrobial agents to prevent colonization. Essential oils (EOs) and their components are gaining increasing interest in the food, cosmetic, and pharmaceutical industries because of their relatively safe status, their wide acceptance by consumers, and their exploitation for potential multi-purpose functional use [11].

Plants of the genus *Mentha* produce a class of natural products known as mono-terpenes (C_10_), characterized by *p*-menthone skeleton. Members of this genus are the only sources for the production of one of the most economically important essential oil, menthol, throughout the world [12]. *Mentha piperita*, commonly called peppermint, is a well-known herbal remedy used for a variety of symptoms and diseases, recognized for its carminative, stimulating, antispasmodic, antiseptic, antibacterial, and antifungal activities [4,13,14]. However, their use for clinical purposes is limited by the high volatility of the major compounds.

Due to their high biocompatibility [15] and superparamagnetic behavior, magnetite nanoparticles (Fe_3_O_4_) have attracted attention to their potential applications especially in biomedical fields [16,17], such as magnetic resonance imaging [18-20], hyperthermia [21], biomedical separation and purification [22], bone cancer treatment [21], inhibition of biofilm development [23,24], stabilization of volatile organic compounds [25], antitumoral treatment without application of any alternating magnetic field [26], drug delivery or targeting [27-33], modular microfluidic system for magnetic-responsive controlled drug release, and cell culture [34].This paper reports a new nano-modified prosthetic device surface with anti-pathogenic properties based on magnetite nanoparticles and *M. piperita* essential oil.

## Methods

### Materials

All chemicals were used as received. FeCl_3_ (99.99%), FeSO_4_·7H_2_O (99.00%), NH_3_(28% NH_3_ in H_2_O, ≥99.99% trace metal basis), lauric acid (C_12_) (98.00%), CHCl_3_ (anhydrous, ≥99%, contains 0.5% to 1.0% ethanol as stabilizer),and CH_3_OH (anhydrous, 99.8%) were purchased from Sigma-Aldrich. Prosthetic device represented by catheter sections were obtained from ENT (Otolarincology), Department of Coltea Hospital, Bucharest, Romania.

### Fabrication of nano-modified prosthetic device

For the fabrication of the nano-modified prosthetic device, we used a recently published method [35] in order to design a new anti-pathogenic surface coated with nanofluid by combining the unique properties of magnetite nanoparticles to prevent biofilm development and the antimicrobial activity of *M. piperita* essential oil.

*M. piperita* plant material was purchased from a local supplier and subjected to essential oil extraction. A Neo Clevenger-type apparatus was used to perform microwave-assisted extractions. Chemical composition was settled by GC-MS analysis according to our recently published paper [36].

Magnetite (Fe_3_O_4_) is usually prepared by precipitation method [37-39]. The core/shell nanostructure used in this paper was prepared and characterized using a method we previously described [40]. Briefly, sodium laurate (C_12_) was added under stirring to a basic aqueous solution of NH_3_, and then FeSO_4_/FeCl_3_ (1:2 molar ratio) was dropped under continuous stirring up to pH = 8, leading to the formation of a black precipitate. The product was repeatedly washed with methanol and separated with a strong NdFeB permanent magnet. The obtained powder was identified as magnetite by XRD. Dimension of the core/shell structure not exceeding 5 nm and their spherical shape were confirmed by TEM analysis. The FT-IR analysis identified the organic coating agent, i.e., lauric acid on the surface of the magnetite nanoparticles. In order to fabricate a modified surface of prosthetic device, core/shell/EO nanofluid was used to create a coated shell. The layer of core/shell/EO nanofluid on the prosthetic device was achieved by submerging the catheter pieces in 5 mL of nanofluid (represented by solubilized core/shell/EO in CHCl_3_ (0.33% *w*/*v*) aligned in a magnetic field of 100 kgf applied for 1 s.

**Figure 1:**
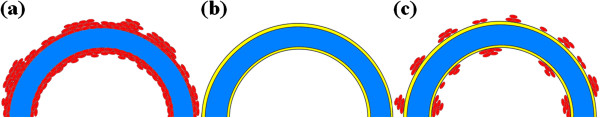
**Biofilm development on the surface of the prosthetic device coated/uncoated with anti-pathogenic nanofluid. (a)**staphylococcal biofilm development on the surface of the prosthetic device, **(b) **nano-modified surface of the prosthetic device, **(c)** inhibition of staphylococcal biofilm development on the nano-modified surface of the prosthetic device.

The catheter pieces were allowed to dry at room temperature. The rapid drying was facilitated by the convenient volatility of chloroform [41]. The coated prosthetic devices were then sterilized by ultraviolet irradiation for 15 min. Figure 1 presents a schematic representation of biofilm development on the surface of the prosthetic device coated/uncoated with anti-pathogenic nanofluid.

### TG analysis

The thermogravimetric (TG) analysis of theFe_3_O_4_@C_12_ and Fe_3_O_4_@C_12_@EO was followed with a Netzsch TG 449C STA Jupiter instrument (Netzsch, Selb, Germany). Samples were screened with 200 mesh prior to analysis, placed in an alumina crucible, and heated at 10 K·min^−1^ from room temperature to 800°C, under the flow of 20 mL min^−1^of dried synthetic air (80% N_2_ and 20% O_2_).

### Biofilm development on nano-modified prosthetic device surface

The adherence of *S. aureus* ATCC 25923 was investigated in six multiwell plates using a static model for monospecific biofilm developing. Catheter pieces of 1 cm with and without coated shell were distributed in plastic wells (one per well) and immersed in the liquid culture medium represented by nutrient broth. The plastic wells were inoculated with 300 μL of 0.5 McFarland microbial suspensions and incubated for 24 h at 37°C. After incubation the culture medium was removed, and the prosthetic device samples were washed three times in phosphate buffered saline (PBS) in order to remove the nonadherent strains and moved into sterile wells. Then, fresh broth was added, the incubation being continued for 72 h. Viable cell counts (VCCs) have been achieved for both working variants (coated and uncoated prosthetic devices) after 24, 48, and 72 h of incubation, respectively, in order to establish the dynamics of the biofilm development and of the inhibitory effect exhibited by the proposed coating system. The adhered cells have been removed from the catheter sections by vortexing and brief sonication, and serial tenfold dilutions ranging from 10^−4^ to 10^−12^ of the obtained inocula have been spotted on Muller-Hinton agar, incubated for 24 h at 37°C, and assessed for VCCs [23,42]. All tests were performed in triplicate.

### Characterization of biofilm development on the surface of nano-modified prosthetic device

After 24, 48, and 72 h of incubation, the samples prepared as described above were removed from the plastic wells, washed three times with PBS, fixed with cold methanol, and dried before microscopic examination. The biofilm development on the surface of coated and uncoated prosthetic devices was visualized using a Hitachi S2600N scanning electron microscope (SEM; Tokyo, Japan) at 25 keV, in primary electron fascicles, on samples covered with a thin silver layer.

## Results and discussion

The increasing occurrence of multiresistant pathogenic bacterial strains has gradually rendered traditional antimicrobial treatment ineffective. The prognosis is worsened by the formation of bacterial biofilms on the biomaterials used in medicine, even if the planktonic cells are susceptible to some antibiotics. Public reports stated that 60% to 85% of all microbial infections involve biofilms developed on natural intact or damaged tissues or artificial devices [43]. These infections are characterized by slow onset, middle-intensity symptoms, chronic evolution, and high tolerance to antibiotics and other antimicrobials [44].

The efficiency of essential oils, polyphenolic extracts obtained from foregoing plants, and their synergic effects as alternative strategies for the treatment of severe infections caused by highly resistant bacteria was tested on the following species: methicillin-resistant *S. aureus*, extended-spectrum beta-lactamases producing *Escherichia coli*, and multiresistant *Pseudomonas aeruginosa*[8]. Previous studies have demonstrated that the mint essential oil (*Mentha* sp.) exhibited synergistic inhibitory effects with low pH and sodium chloride against *Listeria* and inhibited some organisms such as *S. aureus*, *E. coli*, *Candida albicans*, *Acinetobacter baumanii*, *Enterococcus faecalis*, *Klebsiella pneumoniae*, *Salmonella enterica* subsp. *enterica* serotype Typhimurium, and *Serratia marcescens*[45]. The analyzed *M. piperita* EO proved to be rich in β-pinene, limonene, menthone, isomenthol, and menthol. These results are in concordance with reported literature [46,47]. We have suggested before the efficiency of nanosystem-vectored essential oil strategy [23]. The Fe_3_O_4_/C_12_ nanoparticles seem not to be cytotoxic on the HEp2 cell line, which is a great advantage for the *in vivo* use of these nanostructure systems for biomedical applications with minor risks of the occurrence of side effects [48].

**Figure 2:**
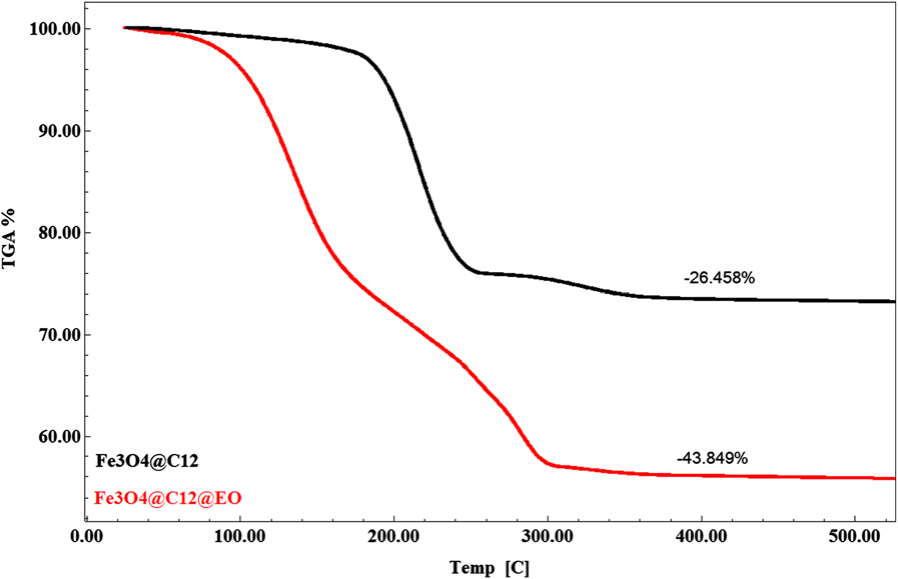
**TGA diagram of Fe**_**3**_**O**_**4**_**@C**_**12 **_**and Fe**_**3**_**O**_**4**_**@C**_**12**_**@EO.**

TG analysis is plotted in Figure 2. The mass loss of EO is up to approximately 170°C, while the mass loss of C_12_ is between 170°C and 375°C. To avoid errors due to overlapping the two regions of weight loss, EO content was estimated as the difference between weight loss for the region at approximately 375°C for both materials, and it is approximately 17.3%.

**Figure 3:**
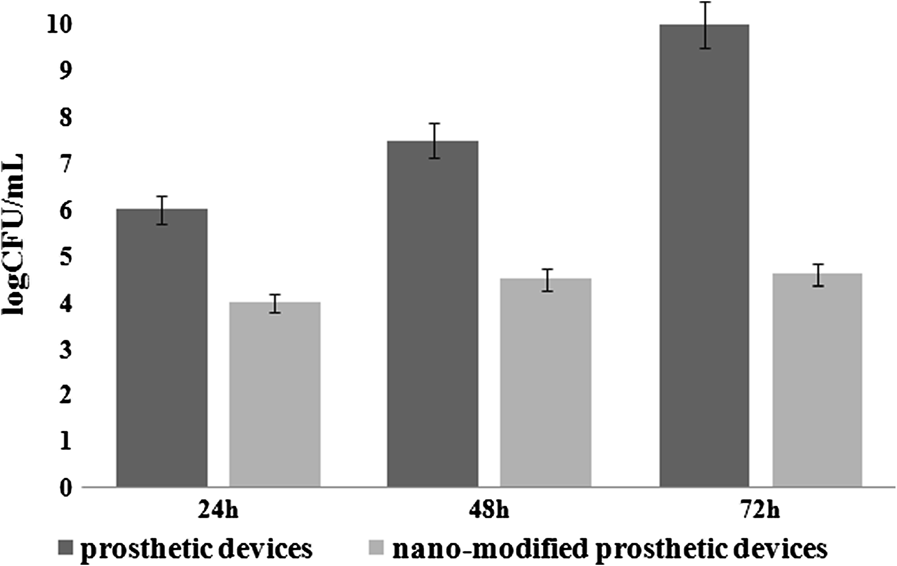
**Viable cell counts recovered from *S. aureus *biofilms developed on the (nano-modified) catheter pieces. **Samples were plated after 24h, 48h and 72h of incubation.

The dynamics of viable cells embedded in the biofilm developed on the catheter device samples showed a significant decrease of the biofilm viable cells, as compared with the uncoated surface (Figure 3). The number of biofilm-embedded cells at 24, 48, and 72 h was almost the same in the case of the coated surface. By comparison, in the case of the uncoated device surface, an ascendant trend of the VVCs was observed for the three analyzed time points. These results suggest that the antibiofilm effect of the obtained coating is remanent, probably due to the gradual release of the essential oil compounds from the coating.

**Figure 4:**
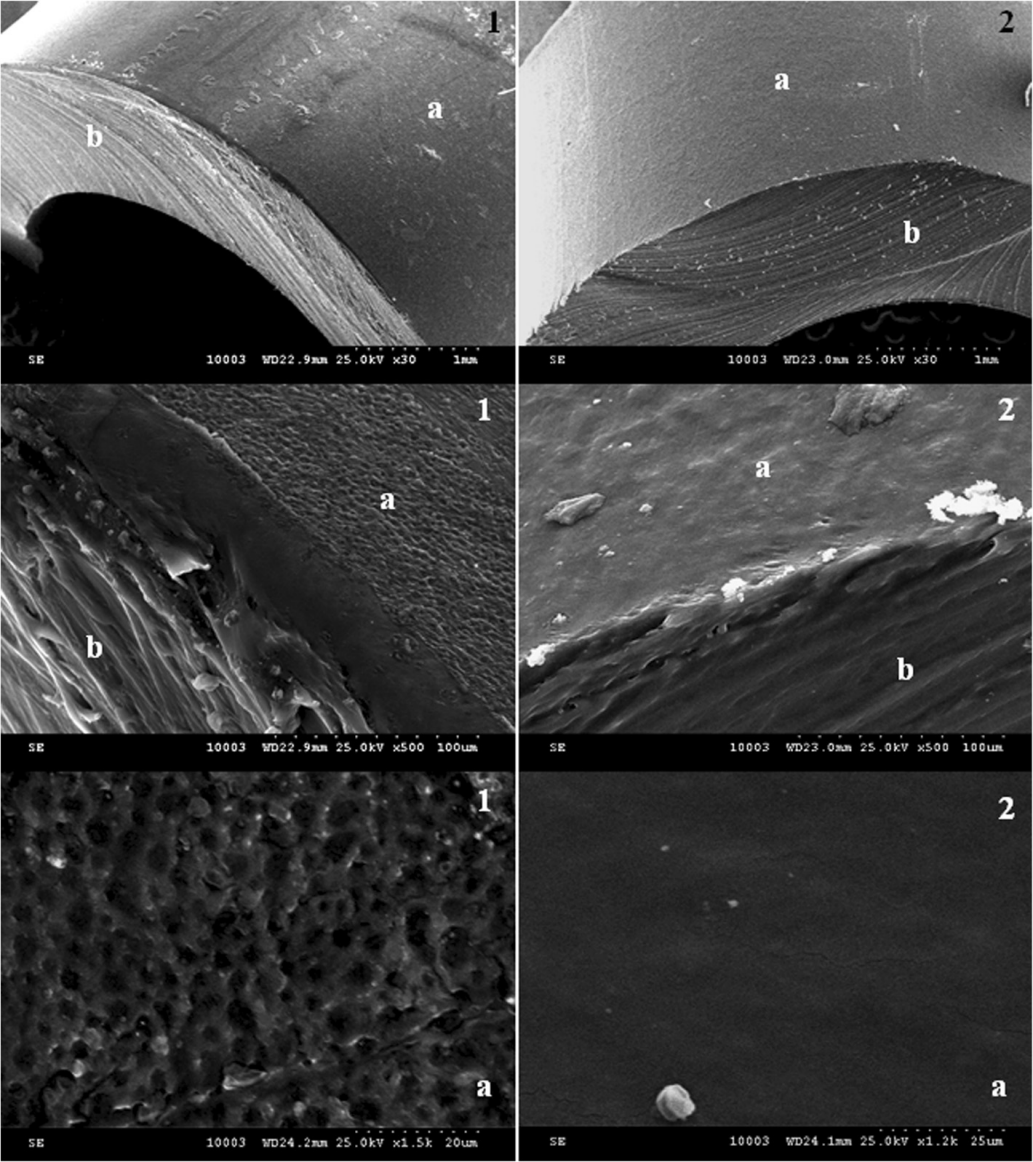
**SEM micrographs of *in vitro *staphylococcal biofilm development on the surface of prosthetic devices. **(**1**) Unmodified prosthetic device sections, (**2**) nano-coated prosthetic device sections, (**a**) surface of the prosthetic device, and (**b**) transversal section of the prosthetic device.

SEM images support the quantitative data, revealing the presence of a well-developed biofilm on the uncoated catheter, as compared with the functionalized one (Figure 4).Taken together, these results are demonstrating that the proposed solution for obtaining a nano-modified prosthetic device is providing an additional barrier to *S. aureus* colonization, an aspect which is very important for the readjustment of the treatment and prevention of infections associated with prosthetic devices.

## Conclusions

In this study, we report the fabrication of a 5 nm core/shell nanostructure combined with *M. piperita* essential oil to obtain a unique surface coating with improved resistance to bacterial adherence and further development of staphylococcal biofilm. The obtained results proved that the proposed strategy is manifesting a dual benefit due to its anti-adherence and microbicidal properties. The microbicidal effect could be explained by the stabilization, decrease of volatility, and controlled release of the essential oil from the core/shell nanostructure. The results reveal a great applicability for the biomedical field, opening new directions for the design of anti-pathogenic film-coated-surface-based core/shell nanostructure and natural products.

## Competing interests

The authors declare that they have no competing interests.

## Authors’ contributions

IA conceived of the study, provided the microbial strain, and drafted the manuscript together with AMG. AMG performed the fabrication of the nano-modified prosthetic devices, obtained the essential oil, and performed the biological analyses. Both authors read and approved the final manuscript.
